# tDCS Task-Oriented Approach Improves Function in Individuals With Fibromyalgia Pain. A Pilot Study

**DOI:** 10.3389/fpain.2021.692250

**Published:** 2021-12-08

**Authors:** Marika Morin, Raphaël St-Gelais, Kossi Épiphane Ketounou, Régis M.-L. d'Assomption, Hassan Ezzaidi, Karen B. P. Fernandes, Rubens A. da Silva, Suzy Ngomo

**Affiliations:** ^1^Laboratoire de recherche Lab BioNR, Physical Therapy Program, Health Sciences Department, Université du Québec à Chicoutimi, Chicoutimi, QC, Canada; ^2^École de Réadaptation, Faculté de Médecine et des Sciences de la Santé, Université de Sherbrooke, Sherbrooke, QC, Canada; ^3^Department of Applied Sciences, Université du Québec à Chicoutimi, Chicoutimi, QC, Canada; ^4^Universidade Norte do Paraná (Unopar), Londrina, Brazil

**Keywords:** tDCS, pain, fibromyalgia, rehabilitation, task-specific training

## Abstract

Fibromyalgia (FM) is a complex pain syndrome accompanied by physical disability and loss of daily life activities. Evidences suggest that modulation of the primary motor cortex (M1) by transcranial direct current stimulation (tDCS) improves functional physical capacity in chronic pain conditions. However, the gain on physical function in people living with FM receiving tDCS is still unclear. This study aimed to evaluate whether the tDCS task-oriented approach improves function and reduces pain in a single cohort of 10 FM. A total of 10 women with FM (60.4 ± 15.37 years old) were enrolled in an intervention including anodal tDCS delivered on M1 (2 mA from a constant stimulator for 20 min); simultaneously they performed a functional task. The anode was placed on the contralateral hemisphere of the dominant hand. Outcome assessments were done before the stimulation, immediately after stimulation and 30 min after the end of tDCS. The same protocol was applied in subsequent sessions. A total of five consecutive days of tDCS were completed. The main outcomes were the number of repetitions achieved and time in active practice to evaluate functional physical task performance such as intensity of the pain (visual analog scale) and level of fatigue (Borg scale). After 5 days of tDCS, the number of repetitions achieved significantly increased by 49% (*p* = 0.012). No change was observed in active practice time. No increase in pain was observed despite the mobility of the painful parts of the body. These results are encouraging since an increase in pain due to the mobilization of painful body parts could have been observed at the end of the 5th day of the experiment. These results support the use of tDCS in task-based rehabilitation.

## Introduction

Fibromyalgia (FM) is a complex syndrome of widespread complaints associated with functional limitation and alteration of the quality of life (1, 2). Pain and physical fatigue have been reported by more than 90% of people living with FM and seem to be important factors in the progression of disabilities (3). FM pain affects negatively some specific articulations, especially the range of motion of the neck-shoulder region. Concerning this fact, some domestic activities, such as dusting kitchen cabinets are particularly painful and limited. The pathogenesis of FM is poorly understood; that is why the International Association for the Study of Pain task force has recently classified (International Classification of Diseases-11) FM clinical condition as a primary pain (5). However, some recent evidences suggest alterations of the central nervous system in the pain FM production mechanism (6). In this context, non-invasive, safe, and well-tolerated neuromodulation approaches seem to be appropriate, like transcranial direct current stimulation (tDCS) (7), as an experimental treatment to reduce pain and improve physical function (8, 9). tDCS physiological effects have not fully been elucidated, but studies suggest that tDCS may modulate the neural circuits responsible for pain processing and perception (10). The cortex is positioned as an entry port for the complex pain-related neural network, and its stimulation seems to interfere with pain signals that originate from the thalamus and other areas in the pain networks of the brain (10). Previous studies suggest that tDCS over the primary motor cortex (M1) reduces chronic pain levels and improves FM-related daily functioning (8, 11, 12). Chronic pain is often accompanied by a high level of kinesiophobia, resulting in an inability to perform household activities (13). Most rehabilitation interventions involving motor training are now task-oriented and varied rather than focusing on the repetition of isolated movements, requiring the use of several joints and movements as in real life. In our recent FM case study, the results of the tDCS analgesia protocol based on a realistic task were encouraging (8). This study aimed to test the same design in a large sample of FM; i.e., to test in more people living with FM whether tDCS improves physical function and reduces pain; and if there is a relationship between pain and performance indicators.

## Methods

### Study Population

Ten chronic FM subjects were recruited and their characteristics are summarized in Table 1. Exclusion criteria were the existence of an uncontrolled health condition, the presence of metallic implants in the skull area, being pregnant, and having any type of physical treatment for <2 months. Written informed consent for participation in the study was obtained from the participants. The local ethic committee of the University of Québec at Chicoutimi approved this study.

**Table 1:** WPI, task performance, and FiRST scores.

**Participant**	**Age**	**WPI score/19**	**Performance day 1**	**Performance day 5**	**FiRST/6**
1	45	4	87	154	5
2	62	18	91	113	6
3	76	17	61	57	6
4	25	16	50	37	5
5	59	9	55	120	6
6	68	19	76	88	6
7	65	12	103	116	6
8	75	12	46	112	6
9	65	14	65	132	6
10	67	14	70	144	5

*Fibromyalgia Rapid Screening Tool (FiRST) is a validated self-completed questionnaire for the detection of fibromyalgia syndrome. It is made up of six items requiring “yes/no” responses and relating to the most relevant clinical features of fibromyalgia. A cut-off score of five items (corresponding to the number of positive items) gives the highest rate of correct identification of FM patients (87.9%), with a sensitivity of 90.5% and a specificity of 85.7% (4)*.

### Study Design

This study included five successive days of evaluation. In the first visit, participants had completed the Fibromyalgia Rapid Screening Tool (FiRST), a validated self-completed questionnaire for detecting fibromyalgia syndrome (4). Moreover, the New Clinical Fibromyalgia Diagnostic Criteria questionnaire was used to provide an index called Widespread Pain Inventory (WPI) which measures the level of the severity according to the number of painful body sites (14). Ten of the nineteen painful body sites are located in the neck-shoulder region (2); it is possible to speculate that this concentration of trigger points in the neck-shoulder region contributes significantly to amplifying the limitations of the upper limbs in the performance of daily physical activities. Consequently, the domestic task in this study was designed to permit the mobilization of that region; it consisted to hang out washcloths on a line as long and accurately as possible without break time during 20 min. Simultaneous, tDCS stimulation was applied. Outcome assessments were done for each subject, before the stimulation (pre-test), immediately after stimulation (post-test 1) and 30 min after the end of stimulation (post-test 2). The same protocol was applied in subsequent experimental sessions.

### Primary Outcome: Domestic Task Performance

A key measure of the task performance was the number of repetitions achieved during the 20 min of a single session. A single repetition was characterized by hanging out one washcloth on a line, which needs a combination of all of the upper extremity movement and skeletal muscle endurance. A second measure was the time in active practice which was defined as the number of minutes per session during which the person was actively practicing tasks without rest periods. The estimate of physical functional performance was based on the average number of repetitions and the time of active practice.

### Secondary Outcomes: Pain and Fatigue

To qualify primary outcome on completion of each session, pain intensity using the visual analog scale (VAS) and fatigue level assessed with modified Borg scale was recorded, before, immediately after the end of the tDCS stimulation and 30 min later. Each subject wrote a daily journal.

### Intervention: Domestic Task in Combination With TDCS Stimulation

**Figure 1:**
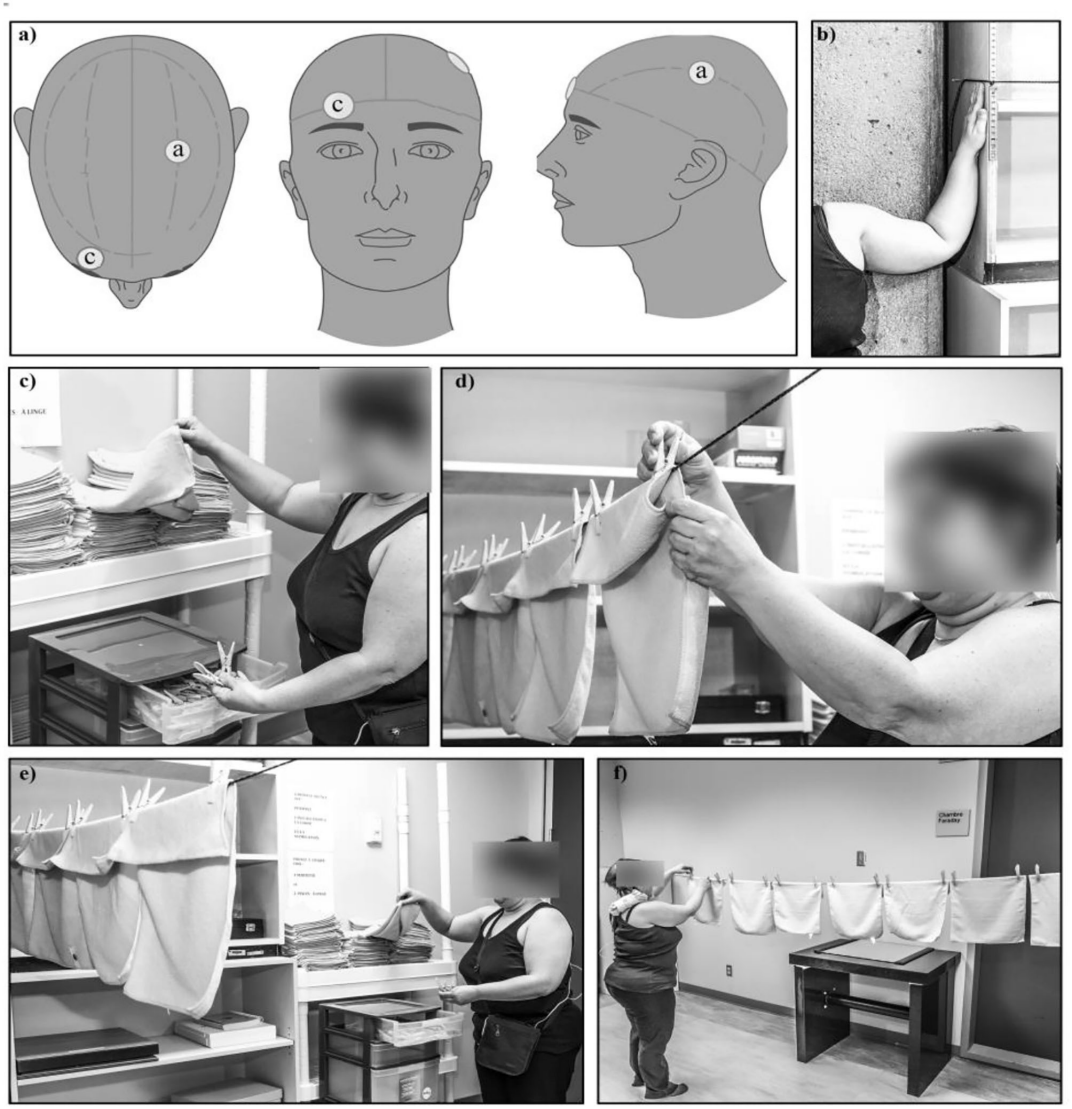
Description of the intervention components. **(a)** An anode (a) was placed above the C3 area of the primary motor cortex and a cathode **(c)** was placed over the contralateral orbit. **(b)** Before the beginning of task performance, the shoulders of participants were moved at 90° abduction, elbows flexed at 90° and the height of the clothesline was adjusted at their fingertips. **(c)** The clothes were placed to a half-meter from the clothesline. The task, in combination with active tDCS, consisted to take one cloth and two pins, then walk to the clothesline and hang it. **(d–f)** The participant has to perform the task as fast as possible considering their pain intensity and may stop any time. Active tDCS and the domestic task were performed simultaneously for 20 min. When the participant reached the end of the clothesline, an experimenter removed the clothes from the line; the participant had to return to the start to continue performing the task. The functional performance consisted of installing as many clothes as possible during the 20 min allowed.

Each participant received five successive daily sessions of tDCS on M1 using a direct current of 2 mA from a constant stimulator for 20 min. The direct current was delivered by two saline-soaked sponge electrodes of 35 cm^2^ localized on the C3 area (anode) and over the supraorbital area (cathode) according to the international system 10/20. The anode was placed on the contralateral hemisphere of the dominant hand (15) (Figure 1a), according to the revised Edinburgh Handedness Inventory (16). The typical initial tDCS tingling sensation provided the signal to start the task. The height of the clothesline was adjusted for each participant as indicated in Figure 1b. The clothes were placed a half-meter from the clothesline. The task consisted to take one cloth and two pins, then walk to the clothesline and hang it (Figures 1c–f). When the participant reached the end of the clothesline, an experimenter removed the clothes from the line; the participant had to return to the start to continue performing the task. Active tDCS and the domestic task were simultaneously for 20 min.

### Statistical Analysis

Descriptive statistics were first calculated for all variables. Then, to verify whether there were changes in measures after tDCS stimulation (pain, fatigue, and performance of task), Friedman two-way analysis of variance was used as follows:

1) Performance of task: (number of cloths or repetitions and time) × days;
2) Pain: (pain intensity before tDCS/after tDCS/30 min after tDCS) × days; and
3) Fatigue: (fatigue before tDCS / after tDCS/30 min after tDCS) × days.

If differences were observed following Friedman two-way ANOVA, *post hoc* analyses were performed using Wilcoxon signed-rank test. To verify the relationship between measures; including WPI scores, a Spearman's R coefficient was calculated. The significance level was set at *p* < 0.05 and all of the statistical analyses were conducted with SPSS version 24 (IBM Corp., Armonk, NY, USA).

## Results

### Descriptive Statistics

All participants (60.4 ± 15.37 years) had an FM diagnosis for at least 7 years; the mean duration of pain was 288.5 (±192.2) months and had at least 5/6 on the FiRST scale. Eight participants had severe pain, one had moderate pain, and one had low pain according to the WPI index score. All were right handed.

The WPI measures the level of the severity according to the number of painful body sites (trigger points) on 19 (14). The performance on day 1 and day 5 of the experiment consisted of installing as many clothes as possible during the 20 min allowed.

### Primary Outcome: Functional Performance

**Figure 2:**
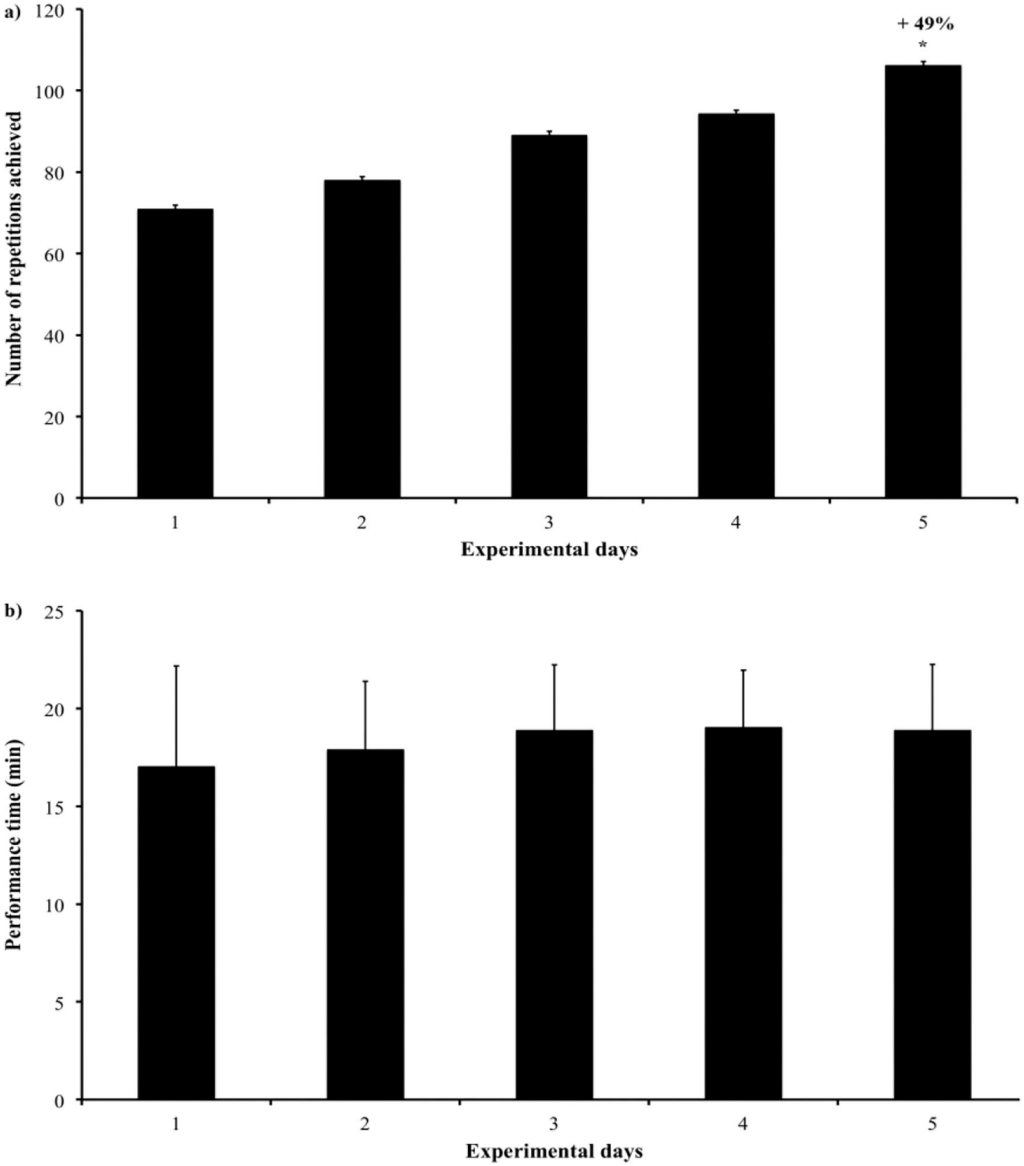
**(a,b)** The number of repetitions achieved during the basic functional task lasted 20 min per day.

The number of achieved repetitions of the basic functional task during 20 min of tDCS increased each day, but a significant gain of 49% was obtained only at the end of the experiment (*p* = 0.012; *z* = −2.521) as shown in Figure 2a. The mean active practice time of the execution of the task was 18.33 min (±0.87), but no significant change in time was observed during experimental days (Figure 2b).

### Secondary Outcomes

Pain intensity did not change throughout the sessions or across days. In people living with FM, the mobilization of the neck/shoulder region during functional tasks should increase the pain level, however, data analysis showed that pain intensity remained stable and suggested a beneficial effect of tDCS stimulation. According to this population, fatigue has a rapid onset and may partly explain the functional disability associated with this condition. The functional task requested and the displacement from home to the laboratory during five consecutive days should result in fatigue, but the absence of change in the level of fatigue may propose a beneficial effect of tDCS (Table 2).

**Table 2:** Pain and fatigue assessment.

**Experimental session**		**Assessment**	**Mean (SD)**	***p*-value (Post-test in comparison to the baseline pre-test, i.e., the data before the start of the experiment)**
1	Pain	Pre-test	5 (2.7)	
		Post-test 1	5.8 (2)	0.260
		Post-test 2	4.2 (2.7)	0.333
	Fatigue	Pre-test	16 (2.8)	
		Post-test 1	13.2 (2.6)	0.157
		Post-test 2	12.3 (3.5)	0.683
2	Pain	Pre-test	4.2 (2.4)	0.575
		Post-test 1	5.6 (1.5)	0.333
		Post-test 2	4.8 (2)	0.878
	Fatigue	Pre-test	12.3(3.5)	0.461
		Post-test 1	13.5 (2.1)	0.655
		Post-test 2	12.7 (3.6)	0.460
3	Pain	Pre-test	4.7 (1.8)	0.767
		Post-test 1	5.3 (1.4)	0.139
		Post-test 2	4.3 (1.8)	0.514
	Fatigue	Pre-test	13.6(2.3)	0.916
		Post-test 1	13 (3.8)	0.655
		Post-test 2	13.4 (3.8)	0.588
4	Pain	Pre-test	4.9 (2.3)	0.553
		Post-test 1	5.3 (1.4)	0.172
		Post-test 2	4.3 (2.1)	0.859
	Fatigue	Pre-test	13 (3.8)	0.450
		Post-test 1	11.5 (0.7)	0.655
		Post-test 2	12.3 (3)	0.705
5	Pain	Pre-test	4.9 (2.8)	0.528
		Post-test 1	4.3(2.6)	0.678
		Post-test 2	5.1 (1.9)	0.677
	Fatigue	Pre-test	9.0 (2.8)	0.223
		Post-test 1	13 (1.4)	0.317
		Post-test 2	11.8 (2.6)	1,000

### Correlation

**Figure 3:**
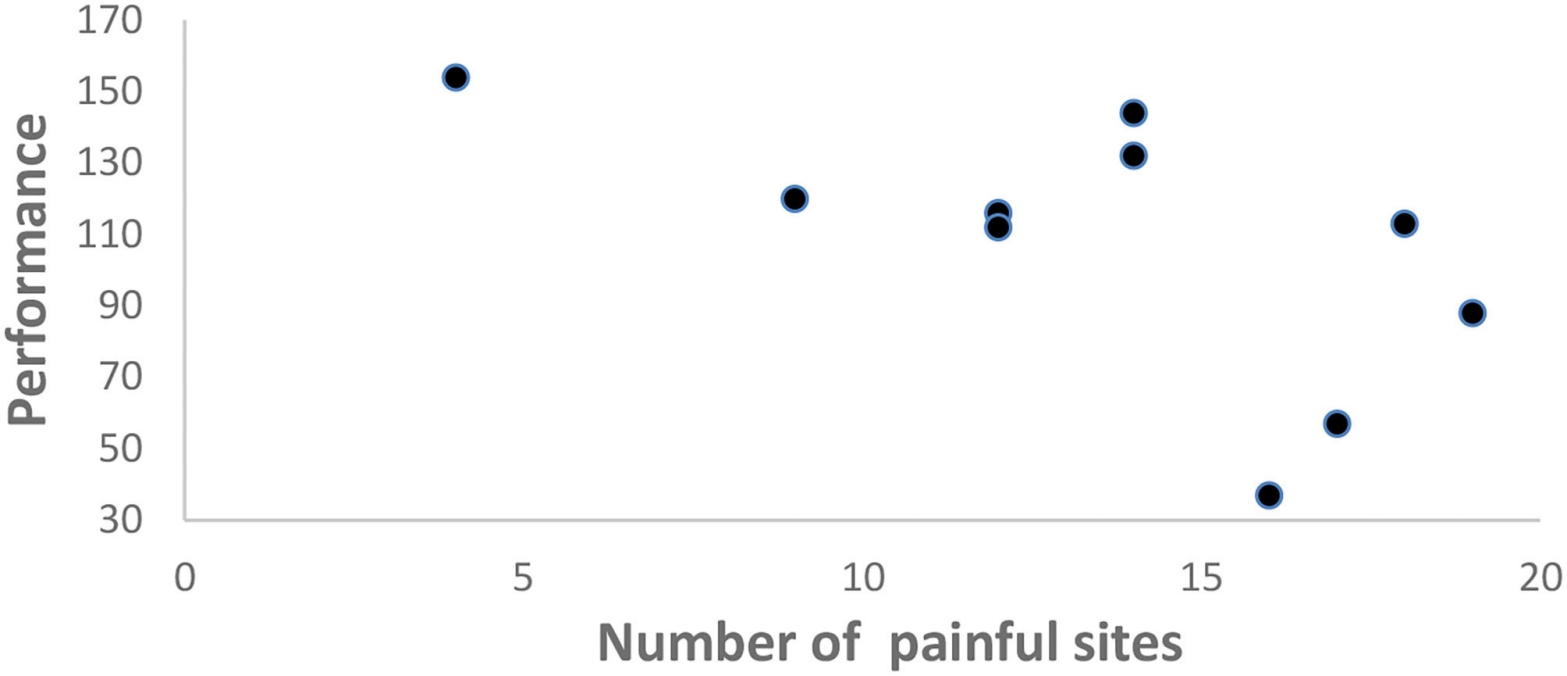
Scatter plot showing the significant negative correlation between WPI score (number of pain sites) and task performance i.e., number of repetitions achieved at the end of the experiment (*p* = 0.049; *r* = −0.634).

The severity of the pain was assessed before beginning the experiment with the WPI score (number of pain sites), which showed that the majority of the sample had a severe pain condition with several body pain sites more than 10 out of 19. A significant negative correlation was found between WPI and the task performance at the end of the experiment (*p* = 0.049; *r* = −0.634) (Figure 3).

No other significant correlation was found.

According to the daily journal, no adverse effects were recorded.

## Discussion

This study investigated whether individuals with FM exhibit changes in physical disability after tDCS stimulation and whether these changes are related to levels of pain and fatigue. Our results showed a significant relationship between the upturn of the function and the liberation of certain body sites by pain after functional tDCS stimulation. Furthermore, the spread of pain, but not its intensity or duration, appears to be a factor related to the function. Our finding related is consistent with previous reports (8, 17, 18). But, FM studies have also shown contradictory results related to the impact of tDCS stimulation in motor actions, highlighting the fragility of tDCS after-effects and suggesting that it may depend on the activity taking place during the stimulation (19). However, this study is the first to combine a domestic functional task with tDCS anodal stimulation, reflecting a possible use of task-oriented tDCS functional stimulation in FM rehabilitation.

Experimental studies indicate a complex scenario potentially relevant to the therapeutic effects of non-invasive brain stimulation techniques for the treatment of neurological disorders such as pain (20). However, the timing of repeated stimulation should be considered concerning the tDCS effect on motor actions. Studies in animals demonstrated that a second tDCS stimulation in the period of after-effects is better for prolonging brain excitability in comparison with a second stimulation occurring 24 h later (21), suggesting better gain in motor capability when inter-interval tDCS stimulation is <24 h. In our paradigm, the interstimulations interval was 24 h, more representative of the clinic process.

Studies have revealed how the effects of tDCS can extend beyond the region underneath the electrodes, thereby influencing global network dynamics, concerning both task-specific activity (22, 23) and pain network (11). In that way, activation of M1 neural circuits in combination with the execution of a physical task would enhance the effects on both motor function and pain. But, our results on pain are in contrast to those Mendonca et al. who observed a significant pain reduction following tDCS stimulation combined with treadmill training. Antidepressants are commonly used to treat chronic pain and 70% of our participants were daily using them. However, medication cure for pain control was an excluding criteria in Mendonca et al.'s recruitment strategy (17). This discrepancy supports Brunoni et al. (24) who reported that treatment-resistant depression with pharmacotherapy was linked with lower tDCS efficacy in pain management. Also, a greater number of trigger points are involved in this study and these sensitive areas may induce a higher neural pain production and possible negative feedback able to reduce the optimal beneficial effects of tDCS on pain and fatigue. The variability in motion events involved to realize different sequences of the requested task is closer to daily life situations.

After encouraging results from our first study, which was a case study, this exploratory pilot study with a more substantial sample highlights the real potential of anodal tDCS treatment paired with a task-oriented approach in FM condition. While the performance status was increasing at the end of the protocol, the pain and fatigue intensity levels did not change. As logically, an increase in pain due to the mobilization of painful body parts could have been observed at the end of the fifth day of the experiment, thus, the maintenance of the level of pain and the increase in mobility are exactly the pathway for pain management, function, and social participation in rehabilitation.

## Limitation

It is well-documented that randomized studies using Sham and active tDCS conditions offer greater methodological robustness. However, this study was conducted in people living with generalized pain of moderate to severe intensity. The protocol with Sham requires a buffer period of 17 days between the two conditions. Each tDCS condition (Sham/active tDCS) requires a minimum of three consecutive days of application. Previous studies on the subject agree on the beneficial effect of active tDCS on pain [see review (25)]. Our original intention was to apply a protocol, as would be the case in task-oriented rehabilitation practice. We, therefore, took care to guarantee participation in the study for five consecutive days. For all these reasons, we chose to use only active tDCS associated with a functional task, with a single baseline measurement before the intervention. Nevertheless, our results suggest functional tDCS as an add-on treatment for FM pain (11). However, these results cannot be assumed to generalize to everyone living with FM pain.

## Conclusion

The functional improvement observed without augmentation of the fatigue or pain is an encouraging result for FM pain condition. These results support the use of tDCS in task-based rehabilitation.

## Data Availability Statement

The datasets presented in this article are not readily available because not authorized by local ethical committee when the study was approved. Requests to access the datasets should be directed to suzy_ngomo@uqac.ca.

## Ethics Statement

The studies involving human participants were reviewed and approved by the local Ethics Committee of the Université du Québec à Chicoutimi. The patients/participants provided their written informed consent to participate in this study.

## Author Contributions

MM, RS-G, and SN: conceptualization. MM, RS-G, KK, Rd'A, HE, and SN: formal analysis. MM, RS-G, and SN: investigation. MM, RS-G, KK, Rd'A, HE, KF, RS, and SN: methodology and writing & original draft. SN: project administration. SN and RS: supervision. KK, Rd'A, HE, KF, RS, and SN: writing & review and editing. All authors contributed to the article and approved the submitted version.

## Conflict of Interest

The authors declare that the research was conducted in the absence of any commercial or financial relationships that could be construed as a potential conflict of interest.

## Publisher's Note

All claims expressed in this article are solely those of the authors and do not necessarily represent those of their affiliated organizations, or those of the publisher, the editors and the reviewers. Any product that may be evaluated in this article, or claim that may be made by its manufacturer, is not guaranteed or endorsed by the publisher.
