# Supplementary figures and images for: tDCS Task-Oriented Approach Improves Function in Individuals With Fibromyalgia Pain. A Pilot Study
Source: Front Pain Res (Lausanne). 2021 Dec 8;2:692250. doi: 10.3389/fpain.2021.692250 (PMC8915725; doi:10.3389/fpain.2021.692250)

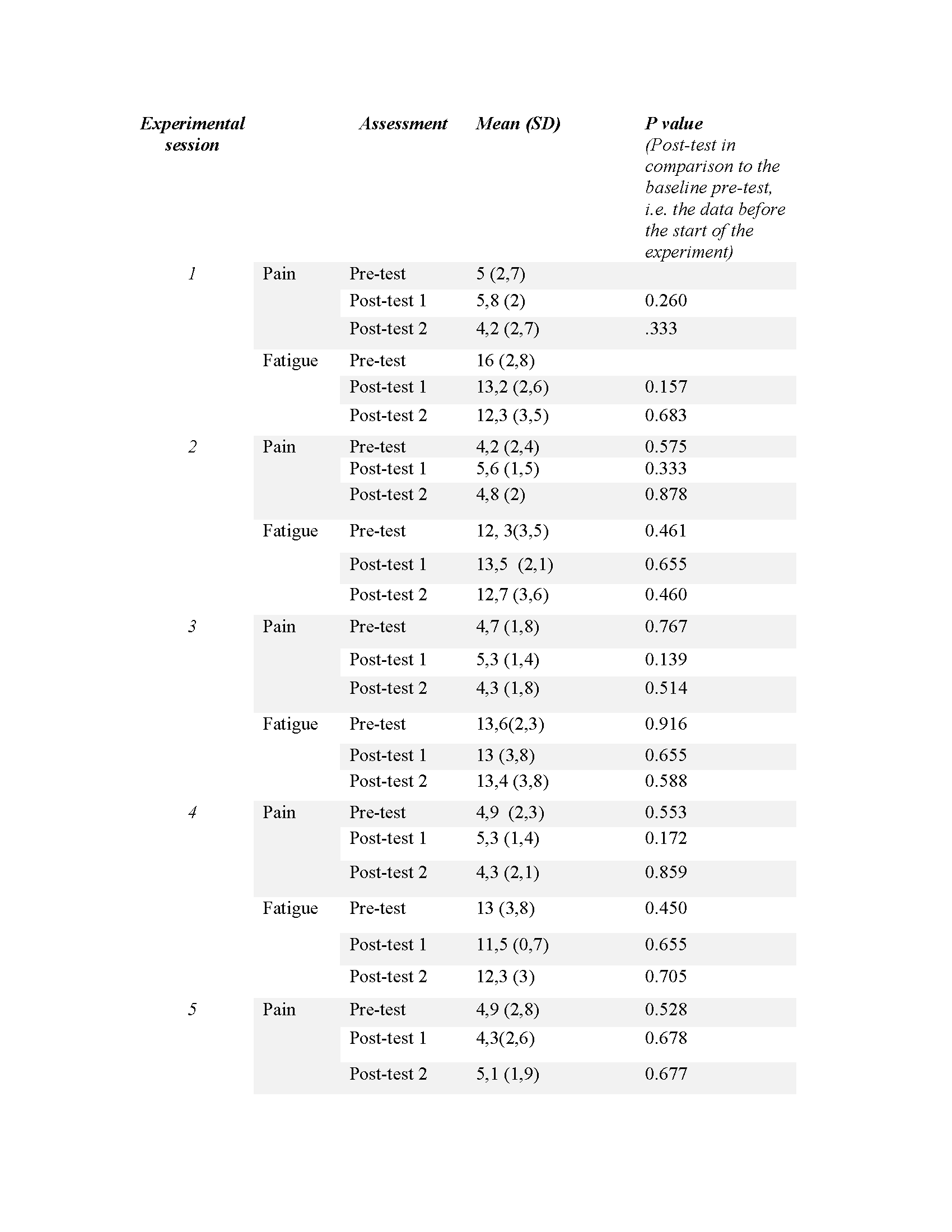

Supplement: Supplementary file 1 [file Image_1.TIF]
